# *Streptococcus pneumoniae* Eradicates Preformed *Staphylococcus aureus* Biofilms through a Mechanism Requiring Physical Contact

**DOI:** 10.3389/fcimb.2016.00104

**Published:** 2016-09-27

**Authors:** Faidad Khan, Xueqing Wu, Gideon L. Matzkin, Mohsin A. Khan, Fuminori Sakai, Jorge E. Vidal

**Affiliations:** ^1^Hubert Department of Global Health at the Rollins School of Public Health, Emory UniversityAtlanta, GA, USA; ^2^National Centre of Excellence in Molecular Biology, University of the PunjabLahore, Pakistan

**Keywords:** *Staphylococcus aureus*, *Streptococcus pneumoniae*, biofilms, physical contact, eradication

## Abstract

*Staphylococcus aureus* (Sau) strains are a main cause of disease, including nosocomial infections which have been linked to the production of biofilms and the propagation of antibiotic resistance strains such as methicillin-resistant *Staphylococcus aureus* (MRSA). A previous study found that *Streptococcus pneumoniae* (Spn) strains kill planktonic cultures of Sau strains. In this work, we have further evaluated in detail the eradication of Sau biofilms and investigated ultrastructural interactions of the biofilmicidal effect. Spn strain D39, which produces the competence stimulating peptide 1 (CSP1), reduced Sau biofilms within 8 h of inoculation, while TIGR4, producing CSP2, eradicated Sau biofilms and planktonic cells within 4 h. Differences were not attributed to pherotypes as other Spn strains producing different pheromones eradicated Sau within 4 h. Experiments using Transwell devices, which physically separated both species growing in the same well, demonstrated that direct contact between Spn and Sau was required to efficiently eradicate Sau biofilms and biofilm-released planktonic cells. Physical contact-mediated killing of Sau was not related to production of hydrogen peroxide as an isogenic TIGR4Δ*spx*B mutant eradicated Sau bacteria within 4 h. Confocal micrographs confirmed eradication of Sau biofilms by TIGR4 and allowed us to visualize ultrastructural point of contacts between Sau and Spn. A time-course study further demonstrated spatial colocalization of Spn chains and Sau tetrads as early as 30 min post-inoculation (Pearson's coefficient >0.72). Finally, precolonized biofilms produced by Sau strain Newman, or MRSA strain USA300, were eradicated by mid-log phase cultures of washed TIGR4 bacteria within 2 h post-inoculation. In conclusion, Spn strains rapidly eradicate pre-colonized Sau aureus biofilms, including those formed by MRSA strains, by a mechanism(s) requiring bacterium-bacterium contact, but independent from the production of hydrogen peroxide.

## Introduction

Two important human pathogens, *Streptococcus pneumoniae* (Spn) and *Staphylococcus aureus* (Sau) persist by forming biofilms in the nasopharynx of healthy humans (Bogaert et al., 2004; Regev-Yochay et al., 2004; Bakaletz, 2007; Chien et al., 2013; Dunne et al., 2013; Shak et al., 2013, 2014; Vidal et al., 2013; Chao et al., 2014). Spn is a common childhood commensal, but also causes otitis media, pneumonia and severe diseases including bacteremia, septicemia, and meningitis (Regev-Yochay et al., 2004; Vidal et al., 2013). Spn, which displays nasopharyngeal carriage rates of up to 90% in children, shifts to a meshed biofilm structure which promotes its persistence in the nasopharynx, increases resistance to antibiotics and acts as a source of planktonic pneumococci, which infiltrate into other parts of the respiratory system (i.e., lungs), bloodstream, and spinal fluid to cause disease (Yarwood et al., 2004; Shak et al., 2013; Vidal et al., 2013; Gritzfeld et al., 2014).

Sau strains, including methicillin-resistant Sau strains (MRSA), colonize the nasopharynx, anterior nares, and skin in 30–50% of healthy individuals, but also produce a variety of infections involving the skin and soft tissue, the bloodstream, the respiratory system, and the skeletal system (Regev-Yochay et al., 2004, 2008; Yarwood et al., 2004; Chien et al., 2013; Dunne et al., 2013; Bhattacharya et al., 2015). Given its location in healthy individuals (i.e., skin), Sau can be easily transmitted in hospital environments, causing a variety of nosocomial infections. Sau-associated nosocomial infections are recognized for their strong ability to form biofilms on abiotic surfaces such as catheters, or indwelling devices. Once a biofilm is established, Sau tolerate concentrations of antimicrobials that would otherwise eradicate planktonic growth (Kiedrowski and Horswill, 2011; Bhattacharya et al., 2015).

Epidemiological studies in children, including those from our laboratory, have demonstrated a negative association for nasopharyngeal carriage of Spn and Sau strains, i.e., children carrying Spn strains in the nasopharynx are less likely to also carry Sau (Chien et al., 2013; Dunne et al., 2013). With the recent introduction of pneumococcal vaccines, this competition for the nasopharyngeal niche has been more evident. For example, a study by Bogaert et al. (2004) that included 3198 children from the Netherlands showed that those vaccinated against Spn experienced a decrease in carriage of Spn vaccine types with a subsequent increase in nasopharyngeal carriage of Sau (Bogaert et al., 2004). Similar evidences were provided by Regev-Yochay et al. (2004) and Chien et al. (2013), in the pre-vaccine era (Regev-Yochay et al., 2004; Chien et al., 2013).

The molecular mechanism(s) behind these epidemiological observations has been investigated without conclusive findings. A study by Regev-Yochay et al. (2006), for example, showed that Spn strains (e.g., Pn20 and TIGR4) interfere with the growth of planktonic cultures of Sau strain Newman by a mechanism likely involving the release of H_2_O_2_ into the supernatant (Regev-Yochay et al., 2006). Killing of Sau planktonic cultures by Spn strains was observed after 6 h of incubation and it was inhibited by the addition of catalase, or by incubating Sau with Spn mutant in the *spx*B gene which encodes for the enzyme producing H_2_O_2_ (i.e., Pn20Δ*spx*B, or TIGR4Δ*spx*B). In contrast, studies using a neonatal rat model of colonization demonstrated that Sau colonizes the nasal passages whether co-inoculated along with TIGR4 or with a TIGR4Δ*spx*B mutant (Margolis, 2009). Moreover, Margolis et al. (2010) showed, using a similar neonatal rat model, that Spn strain TIGR4 coexisted in the nasal epithelium along with Sau, whether Spn or Sau was already colonizing the nasal passages and the other strain was introduced (Margolis et al., 2010). The inconsistencies for the *in vitro* killing vs. co-existence in animal models have not yet been resolved.

Since Sau biofilms have been linked to the persistence of chronic infections that cannot otherwise be eradicated with available antimicrobials (Kiedrowski and Horswill, 2011; Bhattacharya et al., 2015), eradication of Sau biofilms has drawn considerable interest in the last few years. In this study, we have further investigated killing of Sau biofilms using different approaches, including those aimed to eradicate preformed biofilms. We have demonstrated at the ultrastructural level that physical contact is required for efficient killing of Sau by Spn; killing by physical contact eradicated Sau strains, including MRSA strain USA300, within 2 h post-inoculation. In support of these findings, washed bacteria more efficiently killed Sau biofilms than supernatant indicating that the mechanism is more complex than we originally thought. The molecular mechanism, however, warrants further development as complete eradication of Sau biofilms was rapidly achieved.

## Materials and methods

### Bacterial strains and culture media

Spn and Sau strains utilized in this study are shown in Table 1. Spn strains were cultured on blood agar plates (BAP), or BAP with 25 μg/ml gentamicin, whereas Sau strains were grown on salt mannitol agar (SMA) plates or Luria-Bertani agar ([LBA] 1% tryptone [Becton- Dickinson], 0.5% yeast extract, 1% NaCl, and 1.5% agar [Becton-Dickinson]). Todd Hewitt broth containing 0.5% (w/v) yeast extract (THY) was utilized in all experiments.

**Table 1:** **Strains utilized in this study**.

**Strain**	**Description**	**Reference or source**
D39	Avery strain, pherotype CSP1, clinical isolate capsular serotype 2	Avery et al., 1944; Lanie et al., 2007
TIGR4	Invasive clinical isolate, pherotype CSP2, capsular serotype 4	Tettelin et al., 2001
TIGR4Δ*spx*B	TIGR4 with an insertion within the *spx*B gene, *spxB*::*kan-rpsL*+	Regev-Yochay et al., 2006
SPJV01	D39 encoding pMV158GFP, Tet^R^	Vidal et al., 2011
SPJV09	TIGR4 encoding pMV158GFP, Tet^R^	Vidal et al., 2013
GA13499	Phenotype CSP1, capsular serotype 19F	Kindly provided by Dr. Scott Chancey
A66.1	Phenotype CSP2, capsular serotype 3	Benton et al., 1997
*S. aureus* Newman	NCTC 8178, ATCC 13420	Boake, 1956
*S. aureus* ATCC 25923	Clinical isolate, utilized as quality control strain for antimicrobial susceptibility testing	Laboratory stock
*S. aureus* SAJV01	Strain isolated from a post-surgery knee infection in our laboratory at Emory University.	Laboratory stock
*S. aureus* USA300	NRSA384, methicillin-resistant strain isolated from a wound in Mississippi	Centers for Disease Control Prevention, 2003

### Preparation of inoculum for experiments

Inoculum was prepared essentially as previously described (Vidal et al., 2011). Briefly, an overnight BAP (for Spn), or LBA (for Sau), culture was used to prepare a cell suspension in THY broth to an OD_600_ of ~0.08. This suspension was incubated at 37°C in a 5% CO_2_ atmosphere until the culture reached an OD_600_ of ~0.2 (early-log phase). Then glycerol was added to give a final 10% (v/v) and stored at −80°C until used. An aliquot of these stocks was further diluted and plated to obtain bacterial counts (cfu/ml).

### Co-incubation experiments

Experiments were conducted using 8-well glass slide (Lab-Tek), polystyrene 6-well plates and 24-well plates (Corning). Spn and Sau strains were inoculated at a density of ~1 × 10^6^ cfu/ml each in THY and incubated at 37°C in a 5% CO_2_ atmosphere for the indicated time. Control wells were only inoculated with Spn or Sau. Where indicated, bovine liver catalase (Sigma) was added to a final concentration of 1000 U/ml. Planktonic cells were removed, diluted and platted onto BAP or BAP with gentamicin to obtain cfu/ml for Spn or onto LBA or SMA to obtain cfu/ml of Sau. Biofilms were washed once with PBS, mixed with with 1 ml of sterile PBS and sonicated for 15 s in a Bransonic ultrasonic water bath (Branson, Danbury, CT), followed by extensive pipetting to remove remaining attached biofilm bacteria. Biofilms were diluted and platted as above.

### Experiments with preformed Sau biofilms

Sau was inoculated into a 6-well microplate and incubated at 37°C with 5% CO_2_ for 4 h after which planktonic cells were removed and biofilms were washed once with sterile PBS. THY was added to the washed Sau biofilms and then these were inoculated with ~1 × 10^6^ cfu/ml of the early-log phase inoculum, prepared as described above, or with supernatants, planktonic cells, biofilms or washed bacteria obtained from 4 h cultures of Spn. These inoculants were prepared as follows: ~1 × 10^6^ cfu/ml of the early-log phase inoculum was inoculated into 6-well plates and incubated for 4 h. Planktonic cells were then removed, centrifuged, and washed twice with PBS. The supernatant was separated and filter sterilized using a 0.45 μm syringe filter (Puradisc, GE Healthcare, UK). Biofilms were harvested as mentioned earlier and washed twice with sterile PBS. In another set of wells, biofilms were detached by sonication, then both biofilms and planktonic cells were collected by centrifugation, and the pellet was washed twice with PBS. The same amount (~1 × 10^6^ cfu/ml) of washed bacteria, planktonic cells, or biofilms were inoculated into preformed Sau biofilms; an aliquot of supernatant (100 μl) was inoculated as well. Inoculated and control cultures were incubated for 2 h at 37°C with 5% CO_2_ after which bacteria were counted as described.

### Transwell experiments

To physically separate Spn and Sau within the same wells, two chambers were created by installing a Transwell filter device (Corning, NY USA). The Transwell membrane (0.4 μM) creates a physical barrier impermeable to bacteria, but allows passage of small molecules between the two chambers (top and bottom). In some experiments Spn was inoculated in the top chamber and Sau in the bottom chamber, whereas in other experiments bacteria were reversed, i.e., Sau in the top and Spn in the bottom. In control wells, which did not contain the Transwell device, Spn and Sau were inoculated together. Plates were incubated for 4 h at 37°C in a 5% CO_2_ atmosphere and then planktonic and biofilm bacteria were removed from both the top and bottom chamber and counted.

### Confocal microscopy studies

Spn, Sau, or Spn with Sau were inoculated (~1 × 10^6^ cfu/ml each) into 8-well glass slide (Lab-Tek) containing THY and incubated at 37°C in a 5% CO_2_ atmosphere. Planktonic cells were then removed, and biofilms were washed with sterile PBS, after which bacteria were fixed with 2% paraformaldehyde (PFA) for 15 min at room temperature. Fixed biofilms were then blocked with 1% BSA for 30 min at 37°C and incubated first with a rabbit polyclonal anti-Sau antibody (4 μg/ml) (Santa Cruz, Biotechnology Inc.,) for 1 h at room temperature, followed by PBS washes and 1-h incubation with a secondary Alexa-555, labeled goat anti-rabbit antibody (20 μg/ml) (Molecular probes). Then the preparation was washed with sterile PBS and incubated 30 min with rabbit raised anti-Spn antibodies (Staten Serum Institute) that had been previously labeled with Alexa-488 (50 μg/ml) (Molecular Probes) following the manufacturer instructions. In some experiments, Spn strains expressed the green fluorescent protein (GFP), SPJV01 or SPJV09. Stained preparations were finally washed two times with PBS, mounted with ProLong Diamond Antifade mountant with DAPI (Molecular Probes), and analyzed with an Olympus FV1000 confocal microscope. Confocal images were analyzed with ImageJ version 1.49k (National Institutes of Health, USA) or The Imaris software (Bitplane, South Windsor CT).

### Colocalization analysis

The Imaris 8.2 software (Bitplane) was utilized for colocalization analysis. Briefly, the Costes method was utilized to set up a threshold for both the green channel and the red channel in confocal slices of z-stacks images (Costes et al., 2004). The Pearson's coefficient (PC) of colocalized volume was calculated using ranges from −1 to 1 where a PC = −1 indicates a mutually exclusive localization of two signals, PC = 0 random overlap, and PC = 1 indicates perfect colocalization (Costes et al., 2004). Counts of colocalized bacteria and free Sau bacteria was also performed with Imaris 8.2 software.

### Statistical analysis

Statistical analysis presented in this study was conducted using the Mann Whitney *U*-test and the software SigmaPlot Version 12.0 (Systat Software, Inc.).

## Results

### Spn strain TIGR4, but not D39, kills Sau biofilm cells

**Figure 1:**
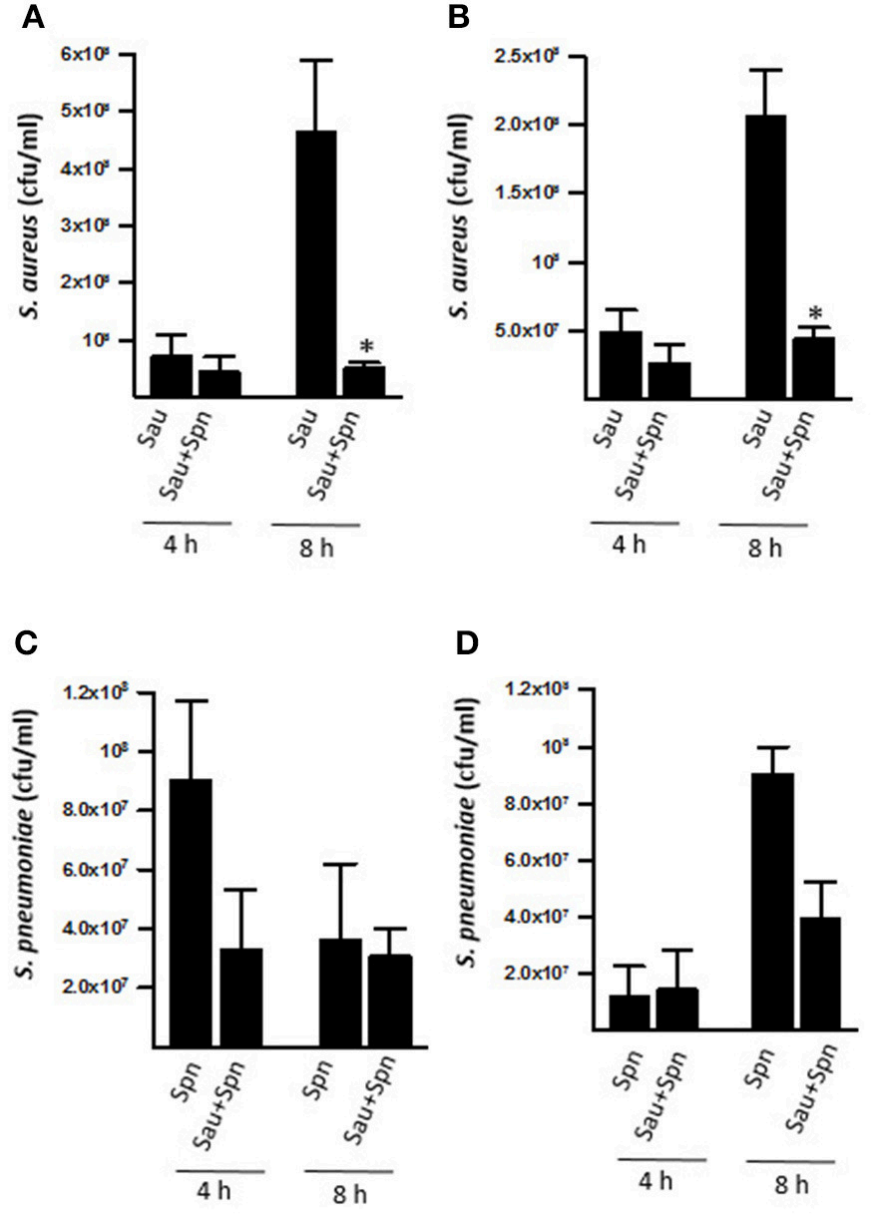
**Spn D39 reduces the population of Sau biofilms**. Sau was inoculated alone (Sau) or with Spn strain D39 (Sau+Spn) in abiotic polystyrene plates **(A,C)** or human pharyngeal cells **(B,D)**. Plates were incubated for 4 or 8 h at 37°C. Planktonic cells were removed, biofilms were harvested, diluted and then plated onto salt mannitol agar plates to obtain Sau biofilm counts (cfu/ml) or blood agar plates with gentamicin to obtain Spn biofilm counts (cfu/ml). Error bars represent the standard errors of the means, calculated using data from at least three independent experiments. ^*^statistical significance (*p* < 0.05) in comparison to wells inoculated only with Sau.

Since epidemiological reports have suggested a negative association between Sau and Spn for nasopharyngeal colonization, we assessed populations of biofilm cells when strains where co-incubated on abiotic surfaces or cultures of human pharyngeal cells. This study showed similar counts of Sau biofilms attached to abiotic surfaces, or pharyngeal cells, whether incubated alone or with Spn strain D39 for 4 h (Figures 1A,B). However, Sau biofilms were significantly reduced, but not eradicated (i.e., completely killed), 8 h post-inoculation (*p* = 0.03) in wells inoculated along with D39 (Figures 1A,B). Bacterial counts of Spn biofilms did not change whether incubated alone or with Sau at 4 or 8 h post-inoculation (Figures 1C,D). A non-statistically significant decrease of Spn biomass was observed, however, when incubated with Sau at 4 or 8 h post-inoculation of abiotic or human cells, respectively.

**Figure 2:**
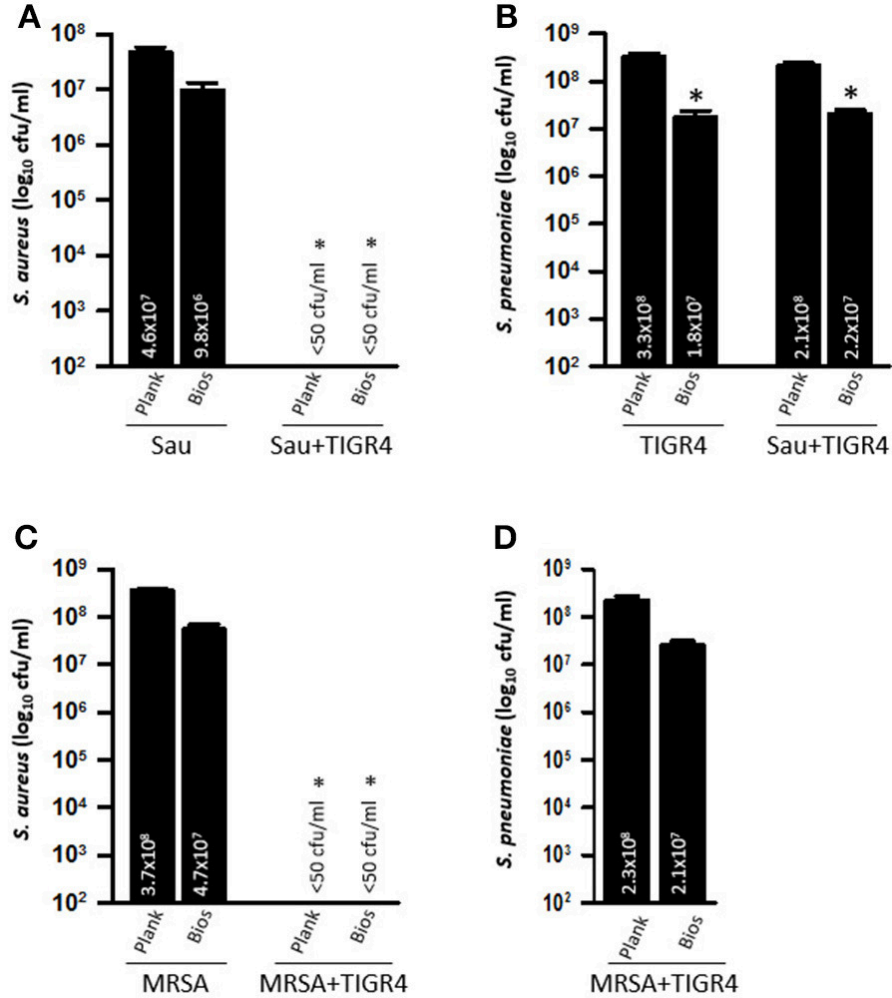
**Spn TIGR4 eradicates Sau biofilms produced by strain Newman and MRSA USA300**. Sau strain Newman **(A,B)** or USA300 **(C,D)** was inoculated alone or with Spn strain TIGR4 and plates were incubated for 4 h at 37°C. Planktonic cells or biofilms were harvested, serially diluted and plated onto salt mannitol agar plates to obtain Sau counts or blood agar plates with gentamicin to obtain TIGR4 counts. Error bars represent the standard errors of the means calculated using data from at least three independent experiments. The median (cfu/ml) is shown inside the bars. ^*^statistical significance (*p* < 0.05) in comparison to wells inoculated only with Sau.

Experiments with another Spn reference strain TIGR4, and Sau strain Newman, were also conducted. Whereas, Sau planktonic cells and biofilms reached, 4 h post-inoculation, a bacterial density of ~4.6 × 10^7^ cfu/ml and ~9.8 × 10^6^ cfu/ml, respectively, Sau planktonic cells and biofilms were eradicated (<50 cfu/ml) when they were incubated with strain TIGR4 for 4 h (Figure 2A). TIGR4 planktonic cells, or biofilms, remained unchanged whether incubated alone or with Sau for 4 h (Figure 2B). MRSA strain USA300, Sau ATCC 25923, SAJV01, were also challenged with TIGR4 for 4 h and eradication of both planktonic and biofilms was similarly observed (Figures 2C,D and not shown).

Since strain D39 produces the competence stimulating peptide 1 (CSP1) and TIGR4 produces CSP2, to further investigate if differences in killing of Sau was due to the quorum sensing pherotype (i.e., CSP1 or CSP2) we inoculated strains GA13499 (pherotype 1) or Spn A66.1 (pherotype 2) along with Sau and the mixtures were incubated for 4 h. Eradication of both Sau planktonic and Sau biofilms by both strains was observed indicating that Sau killing does not depend on the pneumococcal pherotype (not shown).

### Direct contact between Sau and Spn is required for killing of Sau

**Figure 3:**
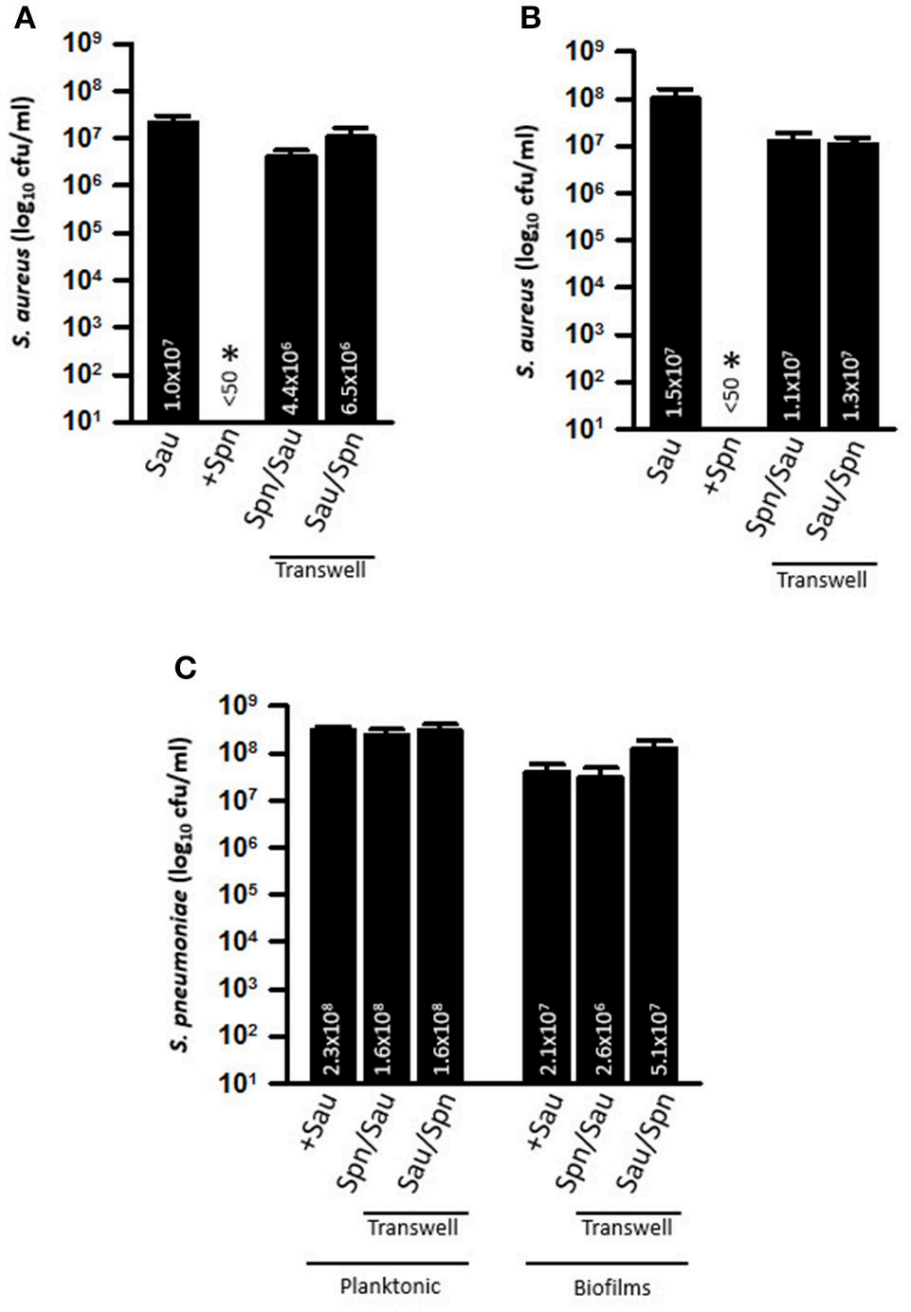
**Efficient Killing of Sau by Spn requires direct contact**. Transwell chambers were installed into 6-well plates and THY was added. TIGR4 was inoculated directly in the Transwell chamber and Sau in the bottom of the well (Sp/Sau), or Sau was inoculated in the Transwell chamber and TIGR4 in the bottom (Sau/Spn). As a control Sau was inoculated alone or with TIGR4 (+Spn). Cultures were incubated for 4 h at 37°C, after which planktonic bacteria **(A)** or biofilms **(B)** were harvested from the Transwell chamber, or from the bottom of the well, serially diluted and plated onto salt mannitol agar plates. **(C)** Planktonic and biofilms were also plated onto BAP plates with gentamicin to obtain Spn counts. Error bars represent the standard errors of the means calculated using data from at least three independent experiments; the median (cfu/ml) is shown inside bars. ^*^Statistical significance (*p* < 0.05) in comparison to wells inoculated with Sau.

To investigate whether Spn biofilm cells or their supernatants were responsible for the observed phenotype against Sau, strains were inoculated into the same wells, but bacteria were separated using a Transwell system device, which has a membrane with a pore size of 0.4 μm. The Transwell device allows the supernatants to flow throughout the well, but separates bacteria inoculated in the top chamber from those inoculated in the bottom of the well. Neither Sau planktonic cells (Figure 3A), nor biofilms (Figure 3B) were killed when TIGR4 was inoculated in the Transwell device and Sau was inoculated in the bottom of the well (i.e., Spn/Sau). Since the Transwell membrane has a smaller diameter than the bottom of the well, in another set of experiments we inoculated TIGR4 in the bottom of the well and Sau was inoculated directly in the Transwell chamber (i.e., Sau/Spn). Once again, TIGR4 was not able to kill Sau planktonic cells or Sau biofilms within 4 h (Figures 3A,B). TIGR4 planktonic cells and biofilms were similar, whether (1) coincubated with Sau (positive control), (2) inoculated in the Transwell chamber and Sau in the bottom or (3) in the bottom of the well when Sau was inoculated in the Transwell chamber (Figure 3C). Experiments were conducted using Transwell devices with different membrane areas (4.67 and 1.12 cm^2^) to account for variations in the volume of culture medium obtaining similar results. Altogether, these experiments demonstrated that physical contact is necessary for Spn to kill Sau.

### Direct killing of Sau by Spn does not require SpxB, but is inhibited by catalase

**Figure 4:**
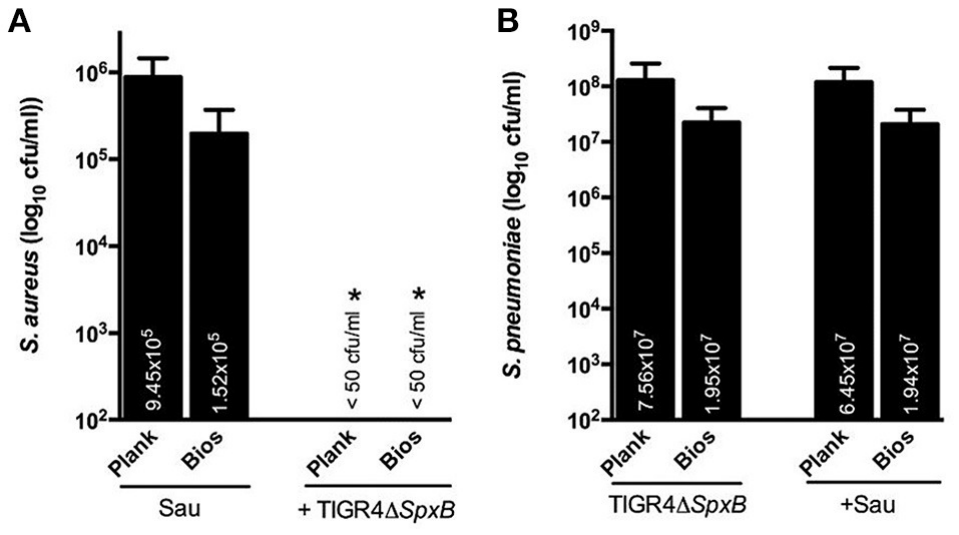
**TIGR4Δ***spx***B mutant eradicate Sau bacteria**. Sau Newman strain was inoculated alone or with TIGR4Δ*spx*B and incubated for 4 h at 37°C. Planktonic cells or biofilms were harvested, serially diluted and plated onto salt mannitol agar plates to obtain Sau counts **(A)** or blood agar plates with gentamicin to obtain TIGR4Δ*spx*B counts **(B)**. Error bars represent the standard errors of the means calculated using data from at least three independent experiments. The median (cfu/ml) is shown inside bars. ^*^statistical significance (*p* < 0.05) in comparison to wells inoculated only with Sau.

Interference of planktonic cultures of Sau by Spn has been demonstrated to occur via hydrogen peroxide, a byproduct of the enzyme SpxB (Regev-Yochay et al., 2006). To investigate whether the observed physical contact-mediated killing requires hydrogen peroxide, we conducted experiments with an isogenic TIGR4Δ*spx*B mutant, which does not produce detectable levels of hydrogen peroxide (Regev-Yochay et al., 2006). As shown in Figure 4A, the hydrogen peroxide TIGR4Δ*spx*B mutant was able to eradicate Sau Newman strain within 4 h of incubation. The population of the isogenic mutant was not affected by co-incubation with Sau (Figure 4B).

**Figure 5:**
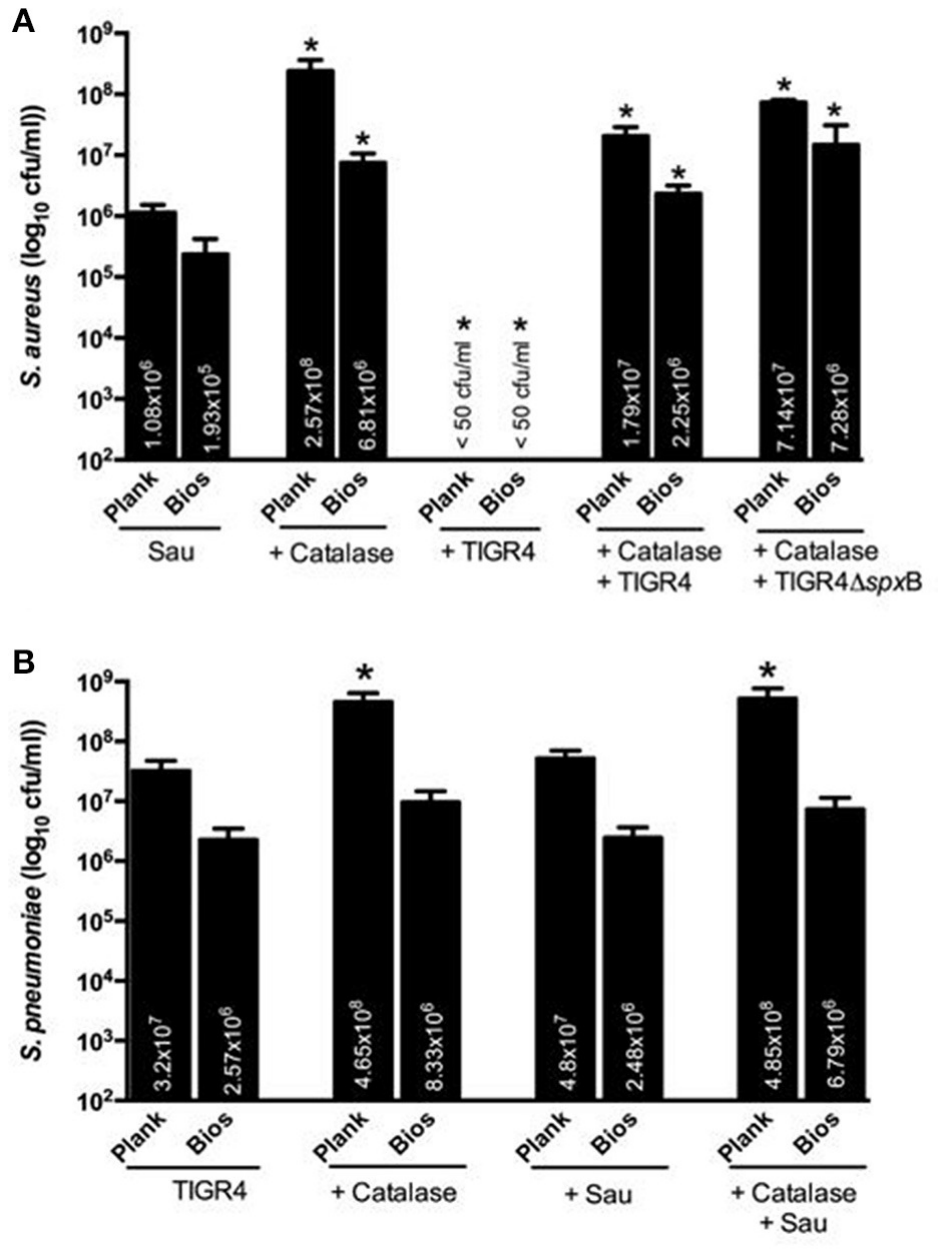
**Catalase inhibits Sau killing by Spn**. Sau Newman strain was inoculated either alone, with catalase, with wt strain TIGR4, with TIGR4 and catalase or with TIGR4Δ*spx*B and catalase and incubated for 4 h at 37°C. Planktonic cells or biofilms were harvested, serially diluted and plated onto salt mannitol agar plates to obtain Sau counts **(A)** or blood agar plates with gentamicin to obtain TIGR4 **(B)**. Error bars represent the standard errors of the means calculated using data from at least three independent experiments. The median (cfu/ml) is shown inside bars. ^*^statistical significance (*p* < 0.05) in comparison to wells inoculated only with Sau.

We next incubated Sau and Spn in the presence of bovine liver catalase. In comparison to co-cultures incubated without catalase, incubation of TIGR4 wt with catalase inhibited killing of Sau (Figure 5A). To investigate whether the inhibitory effect of catalase was separate from its enzymatic activity against H_2_O_2_, the isogenic TIGR4Δ*spx*B mutant, which does not produce H_2_O_2_, was also incubated with catalase and this treatment was enough to render TIGR4Δ*spx*B unable to eradicate Sau bacteria (Figure 5A). Whereas, Spn density was similar whether incubated alone or with Sau (Figure 5B), we noticed that in control wells inoculated only with Sau, or Spn, and incubated in the presence of catalase, the bacterial density of both populations, planktonic and biofilms, significantly increased in comparison to wells incubated without the enzyme (Figures 5A,B).

Together, these experiments demonstrate that SpxB-generated hydrogen peroxide is not involved in the direct-killing of Sau. These experiments also indicate that the inhibitory effect of catalase is due to other changes induced by incubating with the enzyme, which are separate from catalase's enzymatic activity against H_2_O_2_.

### Physical interaction within biofilms formed by Sau and Spn

**Figure 6:**
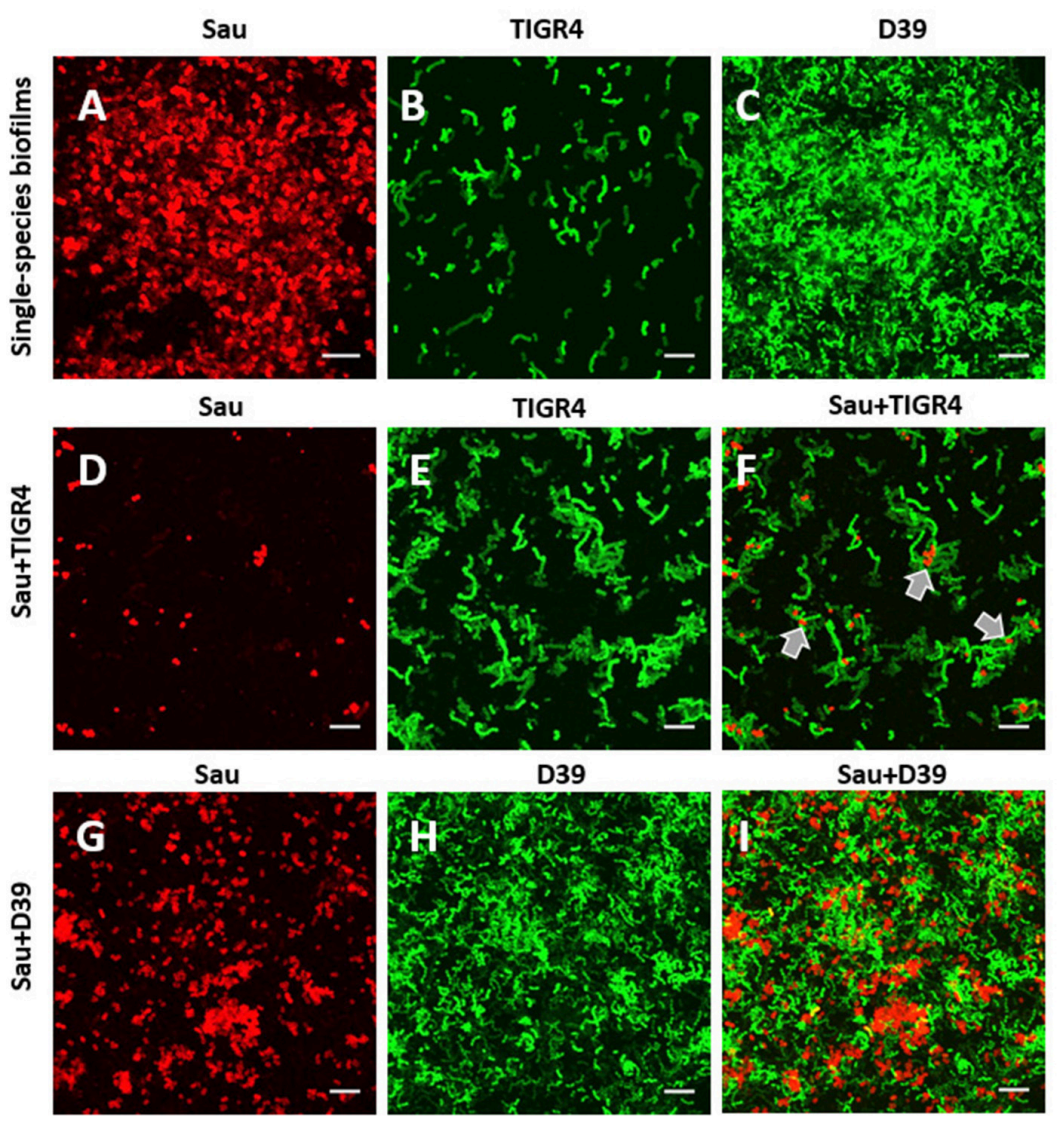
**Confocal studies of Sau coincubated with Spn strains. (A)** Sau, **(B)** SPJV09 (TIGR4), or **(C)** SPJV01 (D39), or mixtures of Sau and SPJV09 **(D–F)** or Sau and SPJV01 **(G–I)** was inoculated into an eight-well slide and incubated for 4 h at 37°C. Biofilms were fixed with 2% PFA and stained with an anti-Sau antibody followed by an Alexa 555-labeled anti-rabbit secondary antibody (red). Spn strains were expressing the green fluorescent protein. Preparations were analyzed by confocal microscopy. A representative xy optical section is shown. Bar = 20 μm. Gray arrows point out areas where Sau and TIGR4 are located.

To gain insights on ultrastructural interactions between TIGR4, or D39, and Sau, we obtained confocal micrographs. At 4 h post-inoculation, control Sau biofilms were robust and covered ~90% of the abiotic substrate (Figure 6A) whereas in wells co-incubated with TIGR4, Sau biofilms were eradicated (Figure 6D). The few Sau cells attached to the substratum appeared to be in close proximity to TIGR4 bacteria suggesting physical interaction between the two species (Figure 6F, arrows). TIGR4 biofilms remained similar whether co-incubated with Sau or incubated alone (Figures 6B,E). Biofilms formed by Sau, when co-incubated with D39, were reduced to ~60% in comparison to control wells (Figures 6G,I). D39 biofilms were similarly observed whether incubated alone or with Sau (Figures 6C,H).

**Figure 7:**
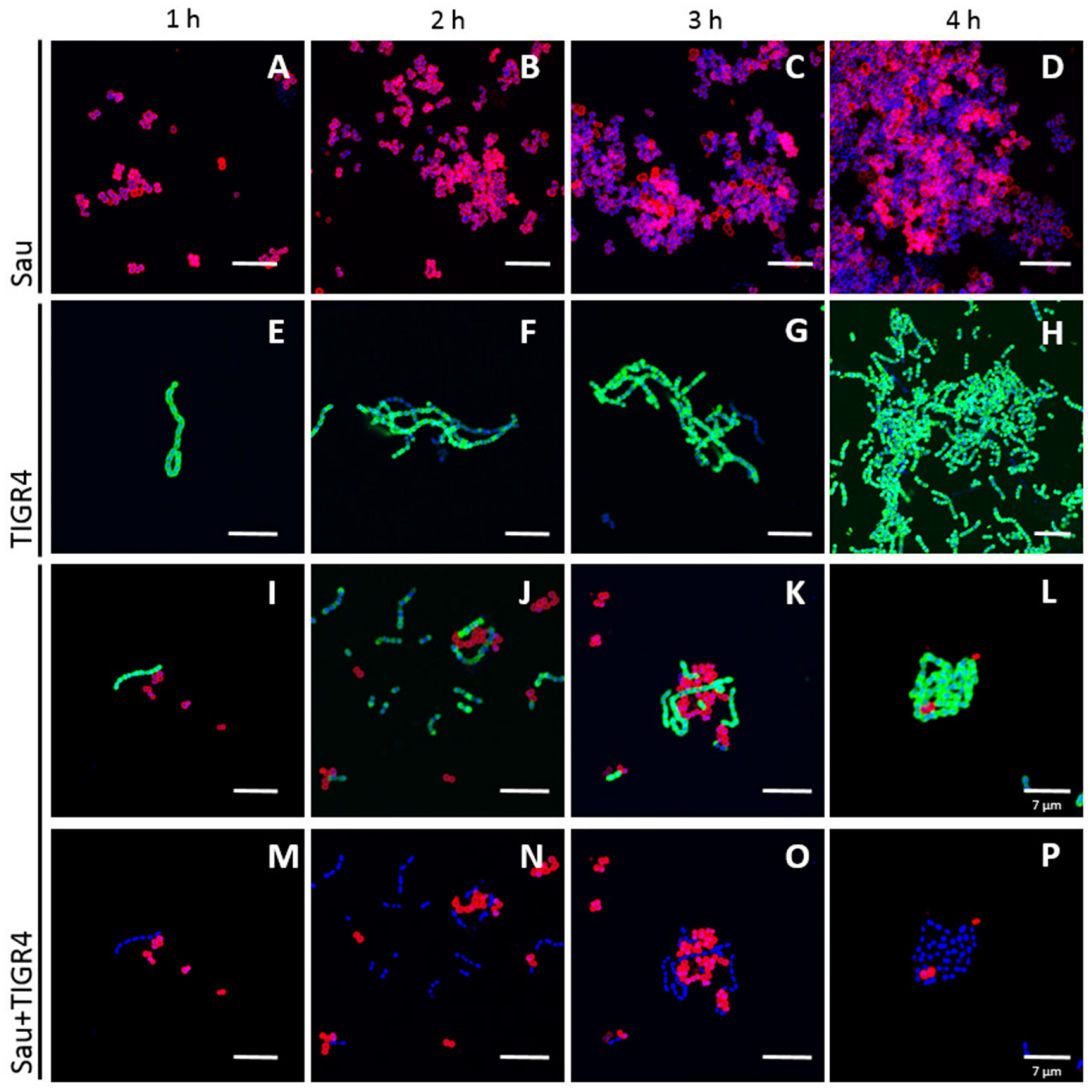
**Time course study of physical interaction between Sau and Spn strains**. Sau **(A–D)**, TIGR4 **(E–H)**, or Sau and TIGR4 **(I–P)** were inoculated into an eight-well slide and incubated for 1, 2, 3, or 4 h at 37°C. Biofilms were fixed with 2% PFA and stained with an anti-Sau antibody followed by an Alexa 555-labeled anti-rabbit secondary antibody (red) and then an anti-Spn antibody labeled with Alexa 488 (green). Bacterial DNA was stained by DAPI (blue). Micrographs were taken by confocal microscopy. Panels show representative xy optical sections (~0.4 μm each). Bar at the right panel is valid for its corresponding horizontal panels. Panels **(I–L)** show the red and green channels while panels **(M–P)** the red and blue channels. Bars = 10 μm, except were indicated (7 μm).

Given that TIGR4 killed Sau and MRSA strain USA300, a time course study was conducted to evaluate physical interactions in detail. As shown in Figures 7A–D, Sau rapidly formed aggregates, i.e., tetrads, at 1 h post-inoculation, which continued growing until forming a bacterial lawn 4 h later. TIGR4 formed chains that aggregated on the bottom of the well (Figures 7E–H) but did not produce, at this time-point, the robust bacterial lawn observed with Sau. When incubated with TIGR4, Sau biofilms were not produced. The few bacteria attached to the bottom were surrounded by TIGR4 (Figures 7I–L).

### Spatial ultrastructural colocalization between Spn and Sau

**Figure 8:**
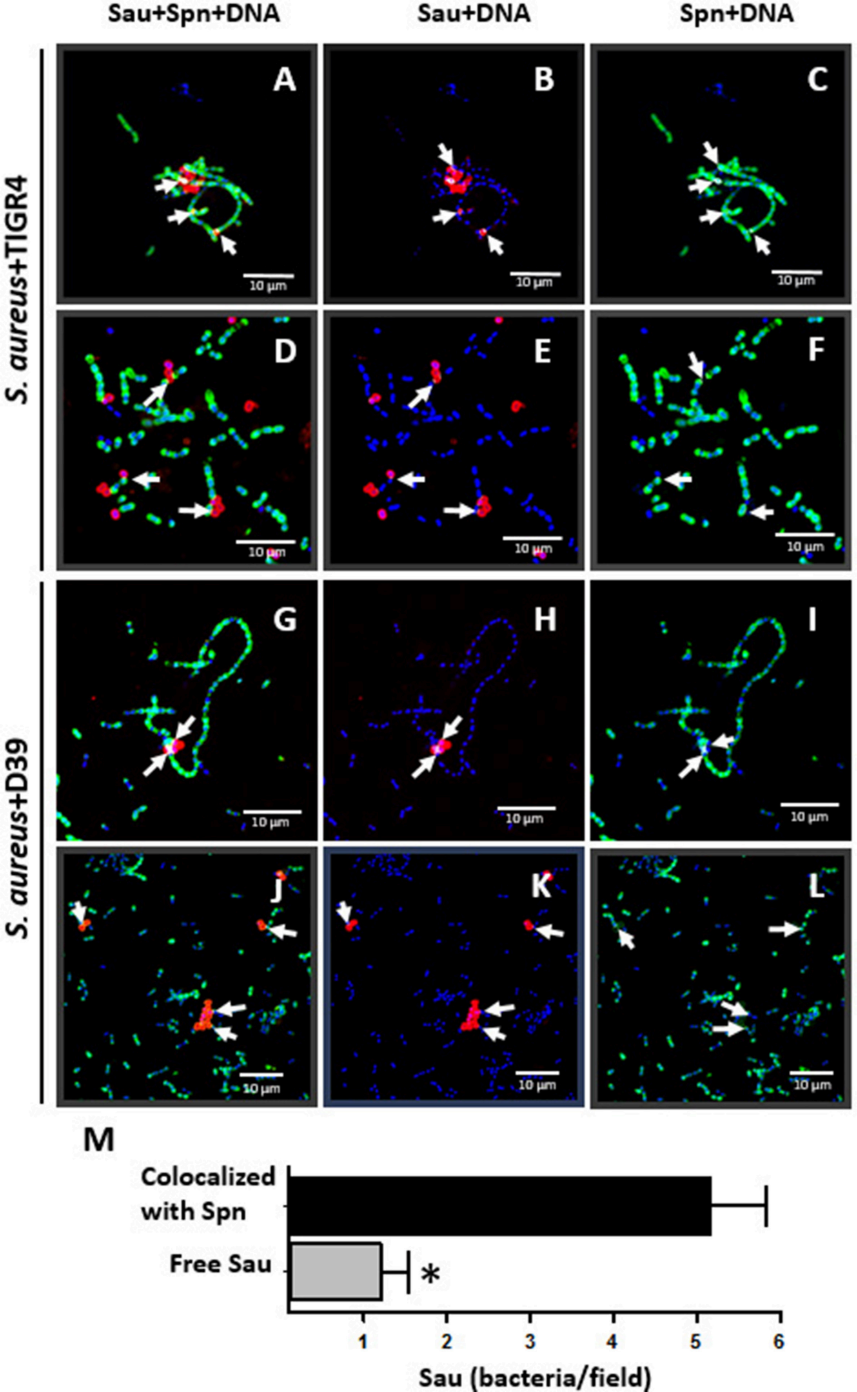
**Colocalization between Sau and Spn**. Sau and TIGR4 **(A–F)** or Sau and D39 **(G–L)** were inoculated together into an eight-well slide and incubated for 1 h **(A–C, G–I)** or 2 h **(D–F, J–L)** at 37°C. Biofilms were fixed with 2% PFA and stained with an anti-Sau antibody followed by an Alexa 555-labeled anti-rabbit secondary antibody (red) and then an anti-Spn antibody labeled with Alexa 488 (green). Bacterial DNA was stained by DAPI (blue). Micrographs were taken by confocal microscopy and analyzed using Imaris software. Panels show representative xy optical sections (~0.4 μm each). Bar = 10 μm at right panels and is valid for its corresponding horizontal panels. Vertical panels show specific channels. Arrows point out areas of colocalization between Sau and Spn. **(M)** Sau colocalized with Spn after 1 h of co-incubation, or free Sau bacteria, were counted in 30 different micrographs. Means were plotted and error bars represent the standard errors. (^*^), statistical significance (*p* < 0.001).

Experiments showed above suggested that Sau and Spn colocalize; to further confirm physical colocalization, we stained the pneumococcal capsule and Sau capsule by fluorescence, and confocal micrographs were analyzed using the Imaris software. As shown in Figure 8, there was a spatial colocalization between Sau and TIGR4 bacteria as early as 1 h post-inoculation. The Pearson's coefficient (PC) of colocalized volume was 0.78, which statistically confirmed true spatial colocalization. TIGR4 surrounded Sau making contact with individual bacterium and those Sau bacteria forming tetrads (Figures 8A–C). Removing the channel of the Spn capsule (green), or Sau capsule (red), allowed us to better visualize specific points of contact (Figure 8, arrows in Sau+DNA and Spn+DNA). Colocalization between Sau and TIGR4 was also observed at 2 h post-inoculation (PC = 0.72) indicating bacteria remained joint (Figures 8D–F). Further analysis of more than 30 confocal micrographs demonstrated that most Sau bacteria are in contact with Spn (mean = 5.16, median = 4), in comparison to those Sau bacteria observed alone (mean = 1.2, median = 0.0; Figure 8M). Whereas, Spn strain D39 did not eradicate Sau biofilms, D39 bacteria were observed colocalizing with Sau at 1 h (PC = 0.73) or 2 h (PC = 0.89) post-inoculation (Figures 8G–L). In most cases a long chain of Spn made contact with tetrads or aggregates of Sau bacteria.

### Pre-colonized Sau is eradicated by TIGR4, but not by strain D39

**Figure 9:**
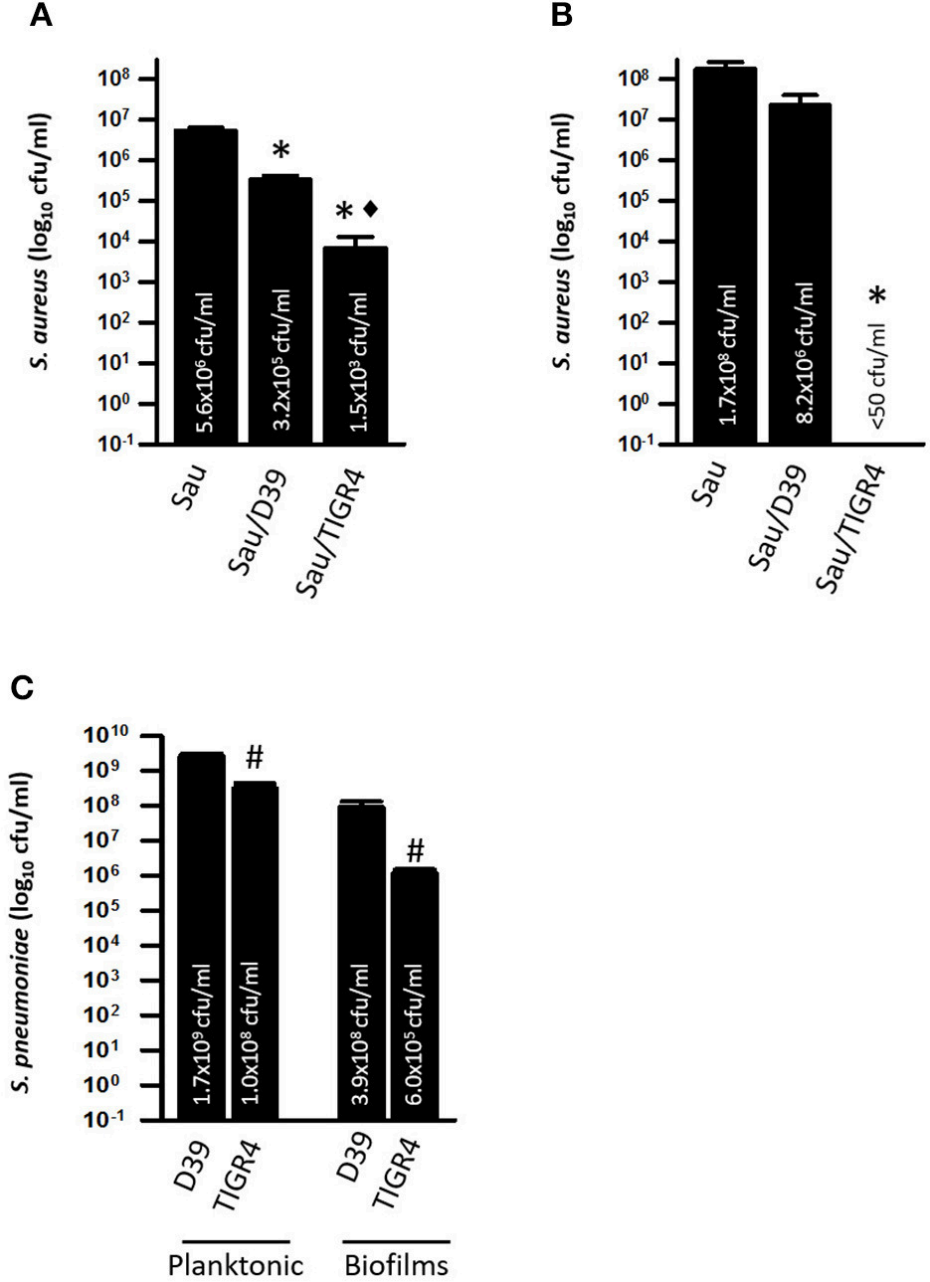
**Spn TIGR4 kills preformed Sau biofilms**. Sau was inoculated in microtiter plates containing THY and incubated for 4 h, after which planktonic cells were removed and fresh THY medium was added. Sau biofilms were left uninoculated (Sau) or co-inoculated with Spn strain D39 (Sau/D39) or TIGR4 (Sau/TIGR4) and incubated for 4 h at 37°C. Biofilms **(A)** or planktonic cells **(B)** were harvested, serially diluted and plated onto salt mannitol agar plates to obtain bacterial counts. **(C)** Dilutions were also plated onto blood agar plates with gentamicin to obtain Spn planktonic and biofilm counts. Error bars represent the standard errors of the means calculated using data from at least three independent experiments; the median (cfu/ml) is shown inside bars. Statistical significance (*p* < 0.05) in comparison to wells inoculated with Sau (^*^), Sau/D39 (♦) or D39 (#).

We then tested whether pre-colonized Sau biofilms could be eradicated by Spn. To assess this, Sau was incubated for 4 h to form biofilms, after which planktonic cells were removed. Early log-phase cultures of D39, or TIGR4 cells, (~1 × 10^6^ cfu/ml) were inoculated into preformed Sau biofilms and then incubated for an additional 4 h period. As seen in Figure 9, pre-colonized Sau biofilms were significantly reduced by incubating with D39, or TIGR4. Furthermore, reduction of pre-formed Sau biofilms by TIGR4 (~1.5 × 10^3^ cfu/ml) was significantly different than Sau reduction produced by incubating with D39 (~3.2 × 10^5^ cfu/ml).

Since biofilms releases planktonic cells into the supernatant, viable planktonic bacteria were also counted. In control wells, Sau planktonic cells released by preformed biofilms reached a density of ~1.7 × 10^8^ cfu/ml (Figure 9B), whereas in pre-formed Sau biofilms inoculated with D39 the population of Sau planktonic cells was reduced, although the reduction was not statistically significant (Figure 9B). Sau planktonic cells (<50 cfu/ml) were eradicated in wells infected with Spn strain TIGR4 (Figure 9B).

Spn counts were obtained in order to investigate if the observed differences in D39 and TIGR4's ability to reduce pre-colonized Sau biofilms and kill planktonic cells was due to an increased population of TIGR4. Both planktonic cells and biofilms were significantly lower in wells inoculated with TIGR4 in comparison to D39 (~200-fold lower) confirming that an increased population was not a factor in the killing of Sau by TIGR4 (Figure 9C).

### Spn bacteria, but not supernatants, efficiently kill Sau pre-colonized biofilms

**Figure 10:**
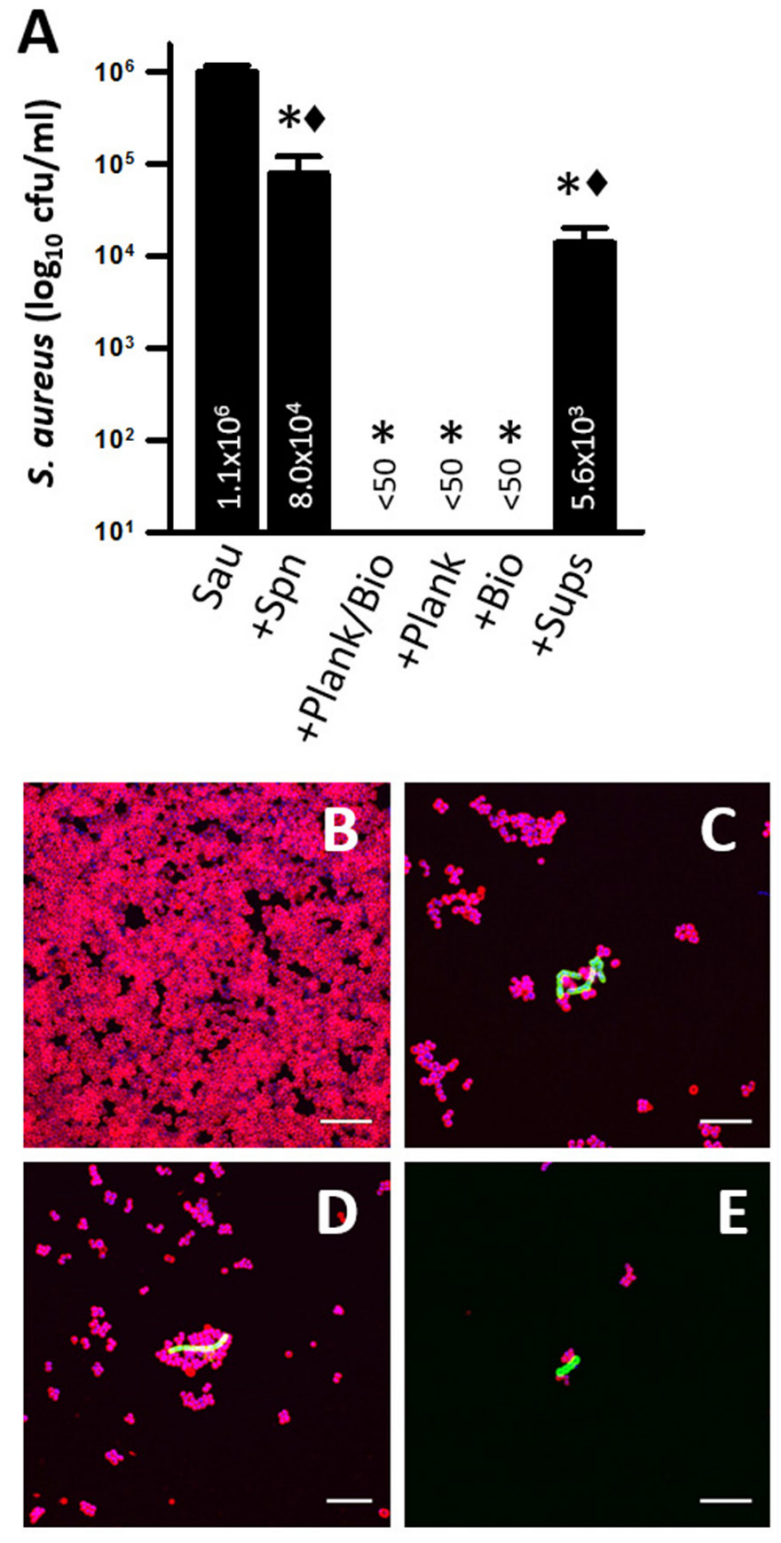
**Washed TIGR4 bacteria rapidly kill preformed Sau biofilms. (A)** Sau was inoculated (Sau) in microtiter plates containing THY and incubated for 4 h, after which planktonic cells were removed and fresh THY medium was added. Another set of wells were inoculated with TIGR4 and incubated for 4 h at 37°C. Planktonic cells, biofilms, or supernatants from this TIGR4 4 h culture were separated as specified in Material and Methods. Preformed Sau biofilms were left uninoculated (Sau), or inoculated with ~1 × 10^6^ cfu/ml of an early-log phase culture of planktonic TIGR4 cells (+Spn), or 4 h cultures of washed bacteria (+Plank/Bio), washed planktonic bacteria (+Plank), washed biofilms (+Bios) or supernatant (+Sup) and incubated for 2 h at 37°C. Cultures were harvested, serially diluted and plated onto salt mannitol agar plates to obtain Sau (cfu/ml). Error bars represent the standard errors of the means calculated using data from at least three independent experiments. Statistical significance in comparison to wells inoculated with (^*^, *p* < 0.004) Sau or (♦, *p* < 0.001) +Plank/Bio. **(B–E)** Sau was inoculated into an eight-well slide and incubated for 4 h at 37°C. Sau Biofilms were challenged with 4 h cultures of washed TIGR4 bacteria and incubated for 30 min **(B)**, 1 h **(C)**, 1.5 h **(D)**, and 2 h **(E)**. At the end of incubation, biofilms were fixed with 2% PFA and stained with an anti-Sau antibody followed by an Alexa 555-labeled anti-rabbit secondary antibody (red) and then an anti-Spn antibody labeled with Alexa 488 (green). DNA was stained with DAPI. Preparations were analyzed by confocal microscopy. A representative xy optical section is shown. Bar = 20 μm.

Our next experiments fractionated TIGR4 cultures into planktonic cells, biofilms and supernatants and evaluated killing of Sau by these fractions. Since inoculating TIGR4 with Sau at the same time eradicated Sau biofilms in 4 h, cultures of TIGR4 were grown for 4 h and then planktonic cells, biofilms and culture supernatant were separated and incubated with preformed Sau biofilms. We hypothesized that Spn from 4 h cultures (i.e., activated cultures) would kill Sau biofilms faster and therefore preformed biofilms were incubated for 2 h. As expected, inoculating preformed Sau biofilms with early log-phase TIGR4 cultures reduced, but did not eradicate, preformed biofilms within 2 h (Figure 10A). However, washed Spn (planktonic+biofilms), planktonic, or biofilms harvested from 4 h cultures eradicated Sau biofilms (Figure 10A). Sterile supernatant from this 4 h culture was only able to reduce Sau biofilms (5.6 × 10^3^ cfu/ml) in comparison with the non-inoculated control (1.1 × 10^6^ cfu/ml).

Experiments were also conducted with supernatants from 6 to 8 h cultures with similar reduction (not shown). Accordingly, confocal micrographs showed robust preformed Sau biofilms, 4 h post-inoculation (Figure 10B), that were significantly reduced within 30 min and 1 h post-inoculation of washed Spn (Figures 10B–D) and completely eradicated within 2 h (Figure 10E). TIGR4 bacteria, however, were not able to recolonize the substrate once Sau biofilms were removed as TIGR4 was only observed attached to the few Sau bacteria, but not attached to the bottom (Figure 10E).

## Discussion

We have demonstrated in this study that TIGR4, and other Spn strains, rapidly eradicated preformed Sau biofilms, including biofilms produced by MRSA strain USA300. To kill Sau biofilms, the pneumococcus required physical contact which was documented by several lines of evidence including confocal microscopy, colocalization experiments, and experiments utilizing a Transwell system to separate both species. The physical contact-mediated killing was very efficient as it completely eradicated a viable lawn of Sau biofilms within 2 h (i.e., viable counts under the limit of detection of 50 cfu/ml).

This efficient mechanism however, was not mediated by production of H_2_O_2_, as an isogenic mutant lacking the enzyme responsible for producing hydrogen peroxide was able to eradicate Sau biofilms and planktonic bacteria. As shown in this work and others (Regev-Yochay et al., 2006), incubating with catalase was enough to inhibit killing of Sau by Spn. We further demonstrated in this study that the inhibitory effect of catalase was separate from its enzymatic activity against hydrogen peroxide, as incubating a TIGR4 isogenic *spx*B mutant, which does not produce H_2_O_2_, with catalase inhibited killing of Sau. Accordingly, Park et al. (2008), showed that catalase produced by Sau strains confers some degree of protection to a challenge with Spn; authors did not utilize, however, a hydrogen peroxide deficient-mutant to verify this protection was directly mediated by its enzymatic activity against H_2_O_2_, as shown in our studies (Park et al., 2008). We hypothesize that incubating Spn with catalase has downstream effects impacting a mechanism that seems to be more complex than originally thought. Future transcriptomic studies should help us to identify, if any, these changes. Changes in bacterial densities, affected by incubating with catalase (Figure 5B), can also be a factor, as bacterial density ratios favoring the pneumococcus are required to eradicate Sau bacteria (discussed below).

Sau strains, including MRSA strains, were the second most common pathogen associated to nosocomial infections in 2011 in the USA accounting for 10.7% of all cases (Magill et al., 2014). In the study by Magill et al. (2014), conducted by the Centers for Disease Control and Prevention (CDC), it was estimated that there were ~721,800 nosocomial infections in 2011. Biofilm-related, device-associated infections, (i.e., central-catheter–associated bloodstream infection, catheter-associated urinary tract infection, and ventilator-associated pneumonia), and surgical-site infections accounted for >47% of those cases (Magill et al., 2014). The majority of Sau nosocomial infections were related to formation of biofilms, i.e., catheter–associated bacteremia. Due to this, efforts are in place to eradicate Sau biofilms and thus decrease hospital-acquired infections and Sau biofilm-related disease.

A number of approaches, other than antibiotics, are now being tested to prevent, or once stablished to eradicate Sau biofilms. Prevention involves the development of new materials that prevent attachment, antibacterial coating, and vaccines (Bhattacharya et al., 2015). Treatment of established Sau biofilms includes matrix degrading enzymes, dispersal triggering agents, small-molecule inhibitors, targeting regulatory molecules, and surgical removal of the focus of infection. Although promising, no single treatment has proven effective to those suffering Sau biofilm disease. Whereas, comparisons were not made with the above mentioned approaches, studies within this work demonstrated complete removal of preformed Sau biofilms within 2 h of incubation with Spn strains TIGR4, A66.1 and GA13499. These observations certainly warrant further investigations and development.

Spn strains can produce two different quorum sensing pheromones, CSP1 and CSP2 (Pestova et al., 1996). The pheromones control competence for transformation (Håvarstein et al., 1995), biofilm formation (Vidal et al., 2013) and lysis of other pneumococci when incubated together, known as fratricide (Steinmoen et al., 2003; Guiral et al., 2005). As shown in our experiments, killing of Sau biofilms was not directly related to the production of a specific quorum sensing pheromone. The possibility exists, however, that a quorum sensing mediated mechanism regulates killing of Sau as our experiments with washed Spn bacteria, mid-log phase (4 h) cultures, killed more rapidly in comparison to early-log phase Spn cultures. Experiments are under way in our laboratories to address the potential role, if any, of quorum sensing in direct killing of Sau biofilms.

A mechanism mediated by the production and release of H_2_O_2_ has been demonstrated for planktonic cultures, and culture supernatants, of Sau strains (Regev-Yochay et al., 2006, 2008). Accordingly, in our study we also observed killing of Sau strains by culture supernatants of Spn (Figure 10), but this was not as efficient as killing of Sau by Spn bacteria. Hydrogen peroxide is a byproduct of the aerobic metabolism produced by pyruvate oxidase, SpxB. Production of H_2_O_2_ has been proposed as the main driver of the negative association between Spn and Sau, as observed in carriage studies (Regev-Yochay et al., 2006, 2008). There is, however, a significant proportion of cocolonization events observed in children (Chien et al., 2013; Dunne et al., 2013). Decreased Spn-mediated killing of some Sau strains was not a factor for the observed cocolonization events, as studies by Regev-Yochay et al. (2008) demonstrated similar bactericidal effect of Sau strains isolated from children co-colonized with pneumococcal strains vs. those only colonized by Sau (Regev-Yochay et al., 2008). Further studies using a neonatal rat model of colonization showed that Spn and Sau can cohabit the nasal passages (Margolis et al., 2010) and that Sau co-colonization rates with Spn TIGR4 wt were similar to those of its isogenic *spx*B mutant (Margolis, 2009). Perhaps levels of H_2_O_2_ in the animal model vs. those obtained in broth cultures are not comparable, which may explain the differences in cocolonization. To our knowledge, levels of H_2_O_2_ produced by Spn in the human nasopharynx or nasal passages in animal models have not been determined. Production of H_2_O_2_ by Spn appears not to be the factor allowing contact-mediated killing of Sau given that, in our study an isogenic spxB mutant was still able to eradicate Sau bacteria. Another streptococci, *S. gordonii*, produces levels of H_2_O_2_ comparable to TIGR4, but is unable to kill Sau (Regev-Yochay et al., 2006). Other lines of evidence indicate that H_2_O_2_ produced by streptococci induces Sau lethal prophages (Selva et al., 2009).

In our study with biofilms, and those conducted with planktonic cultures, killing of Sau required a minimum Spn inoculum of ~1 × 10^6^ cfu/ml to kill the same amount of inoculated Sau bacteria; a reduced Spn challenge, for example ~1 × 10^5^ cfu/ml, will not kill a density of ~1 × 10^6^ cfu/ml of Sau. Physical contact, which we observed in our studies was required for efficient killing, may be a limiting factor for the Spn-Sau required ratio. Another possibility is that a bacterial threshold is required to activate, i.e., by quorum sensing, an efficient killing mechanism which may include the production of enough H_2_O_2_. The need of a bacterial threshold observed in *in vitro* studies may provide an explanation for the cocolonization of Spn and Sau in animal models. For example, in the classic study by Margolis et al. (2010), authors demonstrated cocolonization of <10^4^ cfu of both species, Spn and Sau, in the nasal passages of animals. Perhaps this limited amount of bacteria does not allow for both to reach physical interaction in the nasal microenvironment.

Studies in our laboratory have also recently investigated nasopharyngeal bacterial densities in Tanzanian children cocolonized, or not, with Spn and/or Sau. We, as others in previous studies, demonstrated a negative association for children colonized only with Spn vs. those colonized by both Spn and Sau. Moreover, our study also showed a statistically significant reduction (*p* = 0.03) of Sau density in those children cocolonized with Spn (~1.5 × 10^4^ cfu/ml) vs. those colonized only by Sau (~5.2 × 10^4^ cfu/ml). As per the *in vitro* situation shown in Figures 2, 6 of the current study, nasopharyngeal density of Spn strains in Tanzanian children did not change, whether or not the host was cocolonized with Sau, ~1.5 × 10^6^ cfu/ml vs. ~1.7 × 10^6^ cfu/ml, respectively (Chochua et al., unpublished data; Wu et al., unpublished data).

In conclusion, Spn rapidly eradicates preformed Sau biofilms, including those formed by MRSA strain USA300. The mechanism requires physical contact and a bacterial threshold. Killing of Sau by Spn was not mediated by production of hydrogen peroxide, but it was inhibited by catalase through a mechanism independent of catalase's enzymatic activity against hydrogen peroxide.

## Author contributions

JV, GM, and XW Wrote the paper, conceived research; FK, XW, GM, FS, and MK Performed experiments.

### Conflict of interest statement

The authors declare that the research was conducted in the absence of any commercial or financial relationships that could be construed as a potential conflict of interest.
